# Not just numbers: beyond counting caesarean deliveries to understanding their determinants in Ghana using a population based cross-sectional study

**DOI:** 10.1186/s12884-020-2792-7

**Published:** 2020-02-18

**Authors:** Abdul-Aziz Seidu, John Elvis Hagan, Wonder Agbemavi, Bright Opoku Ahinkorah, Edmond Banafo Nartey, Eugene Budu, Francis Sambah, Thomas Schack

**Affiliations:** 1 Department of Population and Health, University of Cape Coast, Cape Coast, Ghana; 2 Department of Health, Physical Education and Recreation, University of Cape Coast, Cape Coast, Ghana; 3 Neurocognition and Action-Biomechanics Research Group, Faculty of Psychology and Sport Sciences, Bielefeld University, Bielefeld, Germany; 4 The Australian Centre for Public and Population Health Research (ACPPHR), Faculty of Health, University of Technology Sydney, Ultimo, Australia; 5 Ghana Health Service, Abura Dunkwa Health Directorate, Abura Dunkwa, Ghana

**Keywords:** Caesarean, Delivery, Ghana, Women, Obstetric

## Abstract

**Background:**

The increasing rate of caesarean deliveries (CD) has become a serious concern for public health experts globally. Despite this health concern, research on factors associated CD in many low- and -middle countries like Ghana is sparse. This study, therefore, assessed the prevalence and determinants of CD among child-bearing women aged 15–49  in Ghana.

**Methods:**

The study used data from the 2014 Ghana Demographic and Health Survey. The analysis was limited to mothers (*n* = 2742) aged 15–49 , who had given birth in health facilities 5 years preceding the survey. Association between CD and its determinants was assessed by calculating adjusted odds ratios (AOR) with their respective 95% confidence intervals using a binary logistic regression.

**Results:**

The percentage of mothers who delivered their babies through caesarean section (CS) was 18.5%. Using multivariable logistic regression, the results showed that women aged 45–49 (AOR = 10.5; 95% CI: 3.0–37.4), and women from a household that are headed by a female (AOR = 1.3; 95% CI = 1.1–1.7) had higher odds to deliver through CS. Women from the Upper East (AOR =0.4; 95% CI = 0.2–0.7) and Upper West (AOR = 0.4; 95% CI = 0.2–0.8) regions had lower odds to deliver their children through CS. Women with parity 4 or more (AOR = 0.3; 95% CI = 0.2–0.5) had lower odds of CD compared to those with parity 1. Women with female babies had lower odds (AOR = 0.8; CI = 0.7–0.9) of delivering them through CS compared to those with male children.

**Conclusion:**

The percentage of women delivering babies through the CS in Ghana is high. The high rates of CD noted do not essentially indicate good quality care or services. Hence, health facilities offering this medical protocol need to adopt comprehensive and strict measures to ensure detailed medical justifications by doctors for performing these caesarean surgeries.

## Background

Caesarean Section (CS) is one of the obstetric interventions introduced to help decrease maternal and foetal mortality and morbidity [1]. This medical protocol is a surgical procedure in which an incision is made through a mother’s abdomen and uterus to deliver one or more babies, or rarely, to remove a dead foetus [2]. This surgical procedure is viewed as one of the major World Health Organization’s (WHO) medically suggested plan towards improving availability, accessibility, quality, and the use of services for the management and treatment of complications of pregnancy, labour, and delivery [3]. CS is also considered an essential treatment for antepartum haemorrhage, prolonged or obstructed labour, preeclampsia or eclampsia, and intrapartum foetal distress [4]. Nevertheless, a 2008 WHO survey of 373 facilities across 24 countries found that caesareans deliveries (CD) were associated with an increased risk of maternal mortality and serious outcomes for mothers and newborn infants, compared with spontaneous vaginal delivery (VD) [5]. There are 6 to 10 times more complications among women having a CD than a VD, with emergency CDs being two to four times riskier than elective [6].

WHO review from Northern European countries suggests that good maternal and perinatal outcomes are associated with the rate of CS [7]. The WHO concluded that there is no justifiable reason to support caesarean birth rates higher than 15% in any country. A rate between 5 and 15% of births undergoing a CS is optimal and has medical indications for CD [8], and rates above this are unsuitable and unnecessary, imposing financial burden and clinical risks on patients and healthcare systems [9]. A CD rate of less than 5% also indicates the unmet need for skilled delivery service [10].

CS have in recent times been performed upon request for childbirths that could otherwise have been delivered vaginally and these have become a matter of serious concern for public health experts globally [2]. The WHO reports that between 1990 and 2014, the global average CD rate increased from 12.4 to 18.6%, with rates ranging between 6% in low- and -middle-income countries and 27.2% in high income countries [3] and rising at an average rate of 4.4% per year [4]. However, Africa recorded the lowest rate of 7.3%, followed by Asia with 19.2%, Europe recording 25%, and the highest rates of 40.5% from Latin America and the Caribbean [4].

In countries with high prevalence of CD, the factors that contribute to the high rates are low priority of enhancing women’s own abilities to give birth, side effects of common labour interventions, refusal to offer the informed choice of vaginal birth, casual attitudes about surgery and variation in professional practice style, limited awareness of harm more likely with CD, and incentives to practice in a manner that is efficient for providers [1, 4]. Other factors such as type of health facility–public or private [1], age [1, 11, 12], birth order [13–15], birth weight [11], place of residence [1, 16], region of residence [1], socioeconomic status, maternal educational level [1, 17], maternal request [18] and wealth status [1, 17] have all been found to be associated with CS. Most low- and -middle-income countriess (LMICS), however, report CD rates well below the acceptable minimum standard of 5% outlined by the WHO. For many LMICs (e.g., Niger, Ethiopia and Madagascar), the caesarean procedure is scarcely performed, hence the rates of births by CD are around 1.0% [19]. It has been established that in most LMICs, factors including inaccessibility to healthcare, weak healthcare system, inadequate health infrastructure, geographical barriers, cultural factors, poverty, and inadequate human resources are major impediments to providing CD to women who are in dire need of it [20].

Although there have been some studies on CD in Ghana, some of them focused on socioeconomic and demographic factors associated with CD in southern Ghana [14], preference of Ghanaian women for vaginal or CD postpartum [21], validating women’s self-report of emergency CD [18], vaginal birth after a previous CS [22] and clinical indications and feto-maternal outcomes and predictors of CD in Northern Ghana [23]. As far as we know, none of these studies have used a nationally representative sample to assess prevalence and the factors associated with CD among childbearing women in Ghana despite the country recording an overall rate of 16% [24], above the threshold given by WHO. The health system is structured with most of the deliveries initiated in health facilities that do not have the capacity to perform CS and lack ambulances for referrals of clients who need CS in Ghana [25]. Consequently, a lot of pregnant women who undergo emergency CS at referral hospitals have adverse obstetric outcomes as compared to clients who have been booked for parturient [25]. The central focus of this study was, therefore, to determine the prevalence and assess the factors associated with CD among childbearing women in Ghana.

## Methods

### Study setting

The study was carried out in the Republic of Ghana which is located on the West African Coast and has a total land area of 238, 533 km^2^ with 16 administrative regions. Ghana is bordered by three francophone countries; Burkina Faso, Togo and Cote d’Ivoire. These countries lie on the north, East and West of Ghana respectively [24]. In Ghana, from the 2010 population and housing census, there are about 8 main ethnic groups. These are: Akan (47.5%), Mole-Dagbani (16.6%), Ewe (13.9%), Ga–Dangme (7.4%), Gurma (5.7%), Guan (3.7%), Grusi (2.5%), Mande (1.1%), and others (1.4%, [26]. Again, the 2010 census report stated that 51% of the population in Ghana are found in urban areas whereas 49% are in rural areas. There are about 3217 functional health facilities, out of which 4 are teaching hospitals. Additionally, there are 9 regional hospitals, 3 psychiatric hospitals, 11 polyclinics, 59 Christian Health Association of Ghana (CHAG) hospitals, 10 Islamic hospitals, 96 government hospitals, 156 private hospitals, and 22 quasi-government hospitals, 389 maternity homes, and 379 Community–based Head Planning and Services (CHPS) compounds, with majority of these health facilities found in the urban areas [26, 27].

### Data source

The data used for this study were obtained from the 2014 version of the Ghana Demographic and Health Survey (GDHS). The survey uses a standard DHS model questionnaire developed by the Measure DHS programme [24]. The GDHS is a nationwide survey which covers all the then 10 regions of Ghana. The survey is carried out by the GSS and Ghana Health Service with support from ICF International. The key focus of the survey is on child and maternal health in order to provide adequate data to help tract major population and health determinants in Ghana. Specifically, it collects data on fertility, contraceptive use, child health, nutrition, malaria, HIV and AIDS, family planning, health insurance and maternal health; antenatal care, delivery care and post-natal care [24]. For the purpose of this study, women who have given birth in health facilities 5 years preceding the survey were used, thus, 2742 women. Detailed decription of the sampling procedure has been provided in the survey report [24]. Permission to use the data set was given by the MEASURE DHS following the assessment of a proposal. Data set is available to the public at www.measuredhs.org.

### Description and definition of variables

#### Dependent variable

The study used CD as the dependent variable. It was derived from the response to the question “was (NAME) delivered by caesarean, that is, did they cut your belly open to take the baby out?” Responses were categorised Yes = 1 or No = 0.

#### Independent variables

Fourteen independent variables were selected but not chosen arbitrary. The selection was guided by the varied conclusions drawn from some previous studies [1, 11, 12, 14, 28, 29] to be having an association with CD. The independent variables that were used in this study were; maternal age, marital status, education, occupation, wealth status, residence, region, religion, ethnicity, parity (Birth order), number of ANC visits, sex of the baby, size of the baby, and household head’s sex. Maternal age was captured in the DHS as “15–19”, “20 – 24”, “25 – 29”, “20 – 34”, “35 – 39”, “40 – 44”, “45–49”. Marital status was recoded as “Single” and “Living together”. We recoded educational level as “No education”, “Primary education”, “Secondary education” and “Higher education”. Type of Residence was captured as “Urban” or “Rural”. The then 10 regions were also captured in the survey as “Western”, “Central”, “Greater Accra”, “Volta”, “Eastern”, “Ashanti”, “Brong–Ahafo”, “Northern”, “Upper East”, and “Upper West”. Wealth index was measured in quintiles (“poorest”, “poorer”, “middle”, “richer”, “richest”). Ethnicity was coded as “Akan”, “Ga/Adangbe”, “Ewe”, “Guan”, “Mole–Dagbani”, “Grussi”, “Gruma”, “Mande” and “Other”. Parity was recoded as “1”, “2”, “3” and “4+” taking into consideration Ghana’s current total fertility rate of 4.2 [24]. The sex of the baby was coded as “male” and “female”. With respect to occupation, someone was considered as not working if the person was not engaging in any income generating venture; primary occupation was considered as an occupation focusing on the extraction of raw materials including all forms of agriculture; secondary occupation referred to the production industry which adds value to raw materials extracted through primary occupation whilst tertiary occupation involved provision of services. Size of a child at birth was originally coded as “very large”, “larger than average”, “Average”, “smaller than average” and “very small”. These were recoded as “Large” “Average” and “Small”. Considering the recommendations of the WHO [7] and previous studies, a woman should have at least four antenatal visits to prevent negative health outcomes. ANC was therefore coded as “0”, “1–3” and “4 or more”. The place of delivery was recoded as “private” or “public sector”. Women who had home deliveries were not part of the inclusion criteria due to the fact that CD cannot be performed at home [1]. In the Ghanaian context, the public sector includes government hospitals and rural health centres while the private sector includes private hospitals and clinics [30].

### Data analytical strategy

STATA 14.1 for Mac OS (College Station, TX) Statistical Analytic tool was used for the analysis. The outcome variable, CD was coded 0 = “No”, and 1 = “Yes.” Both descriptive and inferential statistics were employed for data analyses. First, a descriptive analysis of socio-demographic characteristics of the participants was carried out and presented as frequencies and percentages in Table 1. Next, a pie chart was used to present results on the prevalence of CD using frequencies and percentages. Third, the association between the independent variables and the outcome variable were presented using a 2 by 2 contingency table and the results presented using chi-square (*χ*^*2*^) and *p* values (see Table 2). The independent variables that were significant at *p* < 0.05 at the bivariate level were used for the multivariable analysis. The results are presented in Table 3 with summary statistics at 95% confidence intervals (CI). Since the outcome variable was a dichotomous variable, the binary logistic regression model was employed. All frequency distributions were weighted while the survey command (svy) in STATA was used to adjust for the complex sampling structure of the data in the regression analyses.

**Table 1:** Socio-demographic characteristics of women aged 15–49 years who gave birth in a health facility in Ghana

Variables	Weighted *n* = 2742	Weighted %
Age		
15–19	61	2.2
20–24	382	14.0
25–29	697	25.4
30–34	688	25.1
35–39	586	21.4
40–44	258	9.4
45–49	69	2.5
Marital status		
Single	209	7.6
Married	1899	69.3
Cohabitating	633	23.1
Education		
No education	407	14.9
Primary	255	9.3
Secondary	1726	63.0
Higher	354	12.9
Occupation		
Not working	444	16.2
Primary	525	19.1
Secondary	359	13.1
Tertiary	1414	51.6
Wealth index		
Poorest	388	14.2
Poorer	438	16.0
Middle	527	19.2
Richer	683	24.9
Richest	706	25.8
Residence		
Rural	1209	43.8
Urban	1543	56.2
Region		
Western	276	10.1
Central	292	10.7
Greater Accra	560	20.4
Volta	189	6.9
Eastern	231	8.4
Ashanti	554	20.2
Brong-Ahafo	250	9.1
Northern	178	6.5
Upper East	141	5.2
Upper West	70	2.5
Religion		
Christian	2188	79.8
Islam	463	16.9
Traditional/spiritual	25	0.9
No religion	65	2.4
Ethnicity		
Akan	1382	50.4
Ga/dangme	189	6.9
Ewe	369	13.5
Guan	54	2.0
Mole-dagbani	472	17.2
Grusi	86	3.1
Gurma	96	3.5
Mande	43	1.6
Other	51	1.9
Parity		
1	567	20.7
2	605	22.1
3	542	19.8
4+	1028	37.5
ANC attendance		
0	15	0.5
1–3	143	5.2
4+	2584	94.3
Type of delivery facility		
Public facility	2425	88.4
Private facility	317	11.6
Sex of baby		
Male	1424	51.9
Female	1318	48.1
Baby size		
Large	1417	51.7
Average	910	33.2
Small	415	15.2
Household head sex		
Male	2093	76.3
Female	649	23.7

Source: Computed from 2014 GDHS

**Table 2:** Bivariate analysis on delivery by caesarean among women aged 15–49 years who gave birth in a health facility in Ghana

Variables	Delivery by caesarean section		Chi-square	
	No	Yes	*χ*^*2*^	*p*-value
Age			37.5	0.000***
15–19	93.7	6.3		
20–24	91.5	8.5		
25–29	83.9	16.1		
30–34	85.6	14.4		
35–39	78.7	21.3		
40–44	81.9	18.1		
45–49	78.7	21.3		
Marital status			4.26	0.119
Never married	82.4	17.6		
Married	83.7	16.3		
Cohabitation	87.0	13.0		
Education			50.4	0.000***
No education	90.3	9.7		
Primary	90.7	9.3		
Secondary	82.5	17.5		
Higher	75.3	24.7		
Occupation			23.6	0.000***
Not working	85.9	14.1		
Primary	89.6	10.4		
Secondary	82.8	17.2		
Tertiary	81.4	18.6		
Wealth index			95.2	0.000***
Poorest	91.3	8.7		
Poorer	88.9	11.1		
Middle	85.9	14.1		
Richer	82.1	17.9		
Richest	71.5	28.5		
Residence			31.4	0.000***
Rural	88.3	11.7		
Urban	80.5	19.5		
Region			65.2	0.000***
Western	81.0	19.0		
Central	80.7	19.3		
Greater accra	74.9	25.1		
Volta	87.4	12.6		
Eastern	83.8	16.2		
Ashanti	77.5	22.5		
Brong Ahafo	86.6	13.4		
Northern	89.2	10.8		
Upper East	91.9	8.1		
Upper West	91.1	8.9		
Religion			3.0	0.393
Christian	83.7	16.3		
Islam	86.5	13.5		
Traditional/spiritual	86.1	13.9		
No religion	82.5	17.5		
Ethnicity			32.1	0.000***
Akan	80.3	19.7		
Ga/Adangme	79.9	20.1		
Ewe	85.5	14.5		
Guan	89.6	10.4		
Mole-Dagbani	88.3	11.7		
Grusi	88.5	11.5		
Gurma	89.8	10.2		
Mande	88.7	11.3		
Other	81.3	18.7		
Parity			14.6	0.002**
1	79.1	20.9		
2	84.9	15.1		
3	85.4	14.6		
4+	86.1	13.9		
ANC attendance			5.7	0.037*
0	85.7	14.3		
1–3	91.0	9.0		
4+	83.9	16.1		
Type of delivery facility			1.6	0.199
Public facility	84.6	15.4		
Private facility	81.5	18.5		
Sex of baby			4.5	0.033*
Male	82.8	17.2		
Female	85.8	14.2		
Baby size			6.0	0.039*
Small	82.0	18.0		
Average	86.6	13.4		
Large	83.5	16.5		
Household head sex			10.3	0.001**
Male	85.5	14.5		
Female	80.0	20.0		

Source: Computed from 2014 GDHS

* *p* < 0.05, ** *p* < 0.01, *** *p* < 0.001

**Table 3:** Multivariable logistic regression on factors associated with CD among women aged 15–49 years who gave birth in a health facility in Ghana

Variable	B	Wald	AOR (95% CI)	*P*-value
Age				
15–19	Ref	Ref	Ref	Ref
20–24	.4572938	0.81	1.6 (0.5–4.9)	0.673
25–29	1.493462	2.22	3.5* (1.2–10.5)	0.015
30–34	1.584549	2.35	3.9* (1.3–11.9)	0.014
35–39	2.237171	3.47	7.5***(2.4–23.6)	< 0.001
40–44	1.684761	3.44	7.9***(2.4–25.9)	< 0.001
45–49	1.088429	3.64	10.5***(3.0–37.4)	< 0.001
Education				
No education	Ref	Ref	Ref	Ref
Primary	−.0980253	−0.44	0.9 (0.5–1.5)	0.674
Secondary	.2760785	1.07	1.2 (0.9–1.8)	0.279
Higher	.3377849	1.65	1.5 (0.9–2.3)	0.065
Occupation				
Not working	Ref	Ref	Ref	Ref
Primary	.0396569	−0.15	1.0 (0.6–1.5)	0.781
Secondary	.0127996	0.06	1.0 (0.7–1.5)	0.910
Tertiary	−.0845761	−0.37	0.9 (0.7–1.3)	0.654
Wealth status				
Poorest	Ref	Ref	Ref	Ref
Poorer	−.2160541	−0.9	0.8(0.5–1.3)	0.406
Middle	−.0459008	−0.17	1.0 (0.6–1.5)	0.897
Richer	.0817143	0.28	1.1(0.6–1.8)	0.594
Richest	.4890988	1.62	1.6(0.9–2.8)	0.049
Residence				
Rural	Ref	Ref	Ref	Ref
Urban	−.0085122	−0.04	1.0(0.7–1.4)	0.953
Region				
Western	Ref	Ref	Ref	Ref
Central	.0428719	0.23	1.1 (0.7–1.6)	0.812
Greater Accra	.0644161	0.33	1.1 (0.7–1.7)	0.780
Volta	−.1135017	−0.51	0.9 (0.5–1.6)	0.625
Eastern	−.0720176	−0.37	0.9 (0.6–1.5)	0.647
Ashanti	.0036808	0.02	1.0(0.7–1.6)	0.849
Brong Ahafo	−.2788066	−1.33	0.7 (0.5–1.2)	0.134
Northern	−.4362156	−1.81	0.6 (0.3–1.0)	0.074
Upper East	−.8430196	−2.89	0.4** (0.2–0.7)	0.003
Upper West	−.6549388	−2.59	0.4** (0.2–0.8)	0.008
Ethnicity				
Akan	Ref	Ref	Ref	Ref
Ga/dangme	−.0516319	−0.35	0.9 (0.6–1.5)	0.625
Ewe	−.2252556	−1.2	0.8 (0.5–1.2)	0.310
Guan	−.2168793	−1.16	0.6 (0.3–1.4)	0.297
Mole-dagbani	.3332827	1.36	1.3 (0.9–1.9)	0.162
Grusi	.027877	0.15	1.0 (0.6–2.0)	0.860
Gurma	−.0290065	−0.15	0.9 (0.5–1.9)	0.887
Mande	.0144463	0.08	1.0(0.4–2.6)	0.800
Other	.0903776	0.68	0.3 (0.6–2.7)	0.446
Parity				
1	Ref	Ref	Ref	Ref
2	−.693029	−3.59	0.5***(0.4–0.8)	< 0.001
3	−.9006956	−4.45	0.4*** (0.3–0.6)	< 0.001
4+	−1.505626	−5.42	0.3***(0.2–0.5)	< 0.001
ANC attendance				
0	Ref	Ref	Ref	Ref
1–3	−.2792714	−0.55	0.6 (0.1–3.1)	0.599
4+	−.0799195	−0.16	0.9 (0.2–3.8)	0.878
Sex of baby				
Male	Ref	Ref	Ref	Ref
Female	−.3017619	−1.99	0.8* (0.6–0.9)	0.041
Baby size				
Small	Ref	Ref	Ref	Ref
Average	−.3214023	−1.9	0.8 (0.6–1.0)	0.055
Large	.1791053	1.14	1.2 (0.9–1.6)	0.248
Sex of household head				
Male	Ref	Ref	Ref	Ref
Female	.2883202	1.97	1.3* (1.1–1.7)	0.027
N			2742	
Pseudo R-sq			0.1	

Source: Computed from 2014 GDHS

* *p* < 0.05, ** *p* < 0.01, *** *p* < 0.001, *Ref* Reference, *AOR* adjusted odds ratio, *CI* confidence interval

## Results

### Maternal socio-demographic characteristics

The survey included the weighted total population of 2742 women who had given birth in health facilities 5 years preceding the survey. Regarding their age distribution, 25% of study participants were in the age range 25–29 years and 30–34 years respectively. For marital status, 7 out of 10 of the women were married. With education, 63% had a secondary level of formal education. Slightly more than half (51.6%) were working in the tertiary sector while 16.2% were non-working mothers whereas 25.8% belonged to the richest quintile of wealth. As far as the region and place of residence were concerned, more than half of the mothers (56.2%) resided in urban areas, while 20.4% were inhabitants of greater Accra region. Akans also constituted half (50.4%) of the sample (see Table 1).

### Prevalence of caesarean delivery

**Fig. 1:**
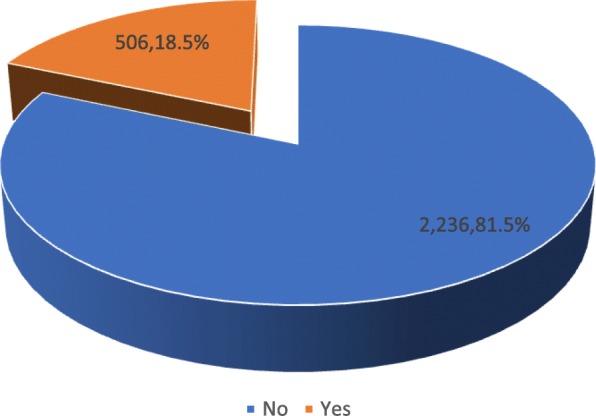
Prevalence of caesarean delivery. Source: computed from 2014 GDHS

Figure 1 shows results on the prevalence of CD among child-bearing women in Ghana. Out of the 2742 women who had given birth in health facilities 5 years preceding the survey, 18.5% delivered their babies through CS and 81.5% had VD.

### Bivariate analysis on delivery by caesarean section

From Table 2, it was shown that there were statistically significant differences in age (*χ*^*2*^ = 37.5, *p* < 0.001), education (*χ*^*2*^ = 50.4, *p* < 0.001), occupation (*χ*^*2*^ = 23.6, *p* < 0.001), wealth (*χ*^*2*^ = 95.2, *p* < 0.001), region (*χ*^*2*^ = 65.2,*p* < 0.001), residence (*χ*^*2*^ = 31.4, *p* < 0.001), ethnicity (*χ*^*2*^ = 32.1, *p* < 0.001), parity (*χ*^*2*^ = 14.6, *p* < 0.01), ANC attendance (*χ*^*2*^ = 5.7, *p* < 0.05), sex of baby (*χ*^*2*^ = 4.5, *p* < 0.05), baby size (*χ*^*2*^ = 6.0, *p* < 0.05), and sex of household head (*χ*^*2*^ = 10.3, *p* < 0.01) and CD. The results further revealed that 21% of the respondents aged 35–39 and 45–49 delivered their children through CS. Approximately, a quarter 24.7% of the respondents with higher level of education delivered their children through CS, 28.5% of mothers who delivered through CS were in the richest domain, 25.1% were in greater Accra region. It was also found that 20.2% were Ga/Adagmes, 20.9% had parity 1, 16.1% were those who had 4 or more ANC visits (see Table 2).

### Multivariable analysis

Using multivariable logistic regression analysis, the data showed that women aged 45–49 had higher odds of giving birth by CS compared to women aged 15–19 years (AOR = 10.5; 95% CI: 3.0–37.4), and women from household that are headed by females (AOR = 1.3; 95% CI = 1.0–1.7) compared to women in households headed by males. Women from the Upper East (AOR =0.4; 95% CI = 0.2–0.7) and Upper West (AOR = 0.4; 95% CI = 0.2–0.8) regions were less likely to deliver children by CS compared to women from the Western region. Similarly, the odds of CD decreased with an increase in parity. For example, women with parity 3 (AOR = 0.4; 95% CI = 0.3–0.6), 4 or more had about 0.3 lower odds of CD deliveries (AOR = 0.3; 95% CI = 0.2–0.5) compared to those with parity 1. Women with female babies had lower odds of CD (AOR = 0.8; CI = 0.7–0.9) compared to those with male children (see Table 3).

## Discussion

CS is a mechanism to save both the mother and the baby. However, delay in deciding for it may be detrimental for both. Nonetheless, premature and wrong decision opting for CD may increase maternal and foetal morbidity and mortality [31]. This paper sought to assess the factors associated with CD among childbearing women in Ghana. This present study found that 18.5% of women who delivered in health facilities 5 years preceding the survey delivered their babies through CS, a figure that exceeds the threshold of 5–15% recommended by WHO [7]. This corroborates the findings in previous studies in low- and -middle income countries such as Pakistan [1], Egypt, [11], Bangladesh [12], Ethiopia [31], India [32], Jordan [33] and Tanzania [34]. An improved propensity toward available medical interventions and continued discouragement of VD after previous CS may have added to the recent increase in CS rates as noted in other countries [35]. Despite the fact that the study was not designed to measure the effect of changes in other maternal characteristics (e.g. Maternal requests), other determinants such as temporal changes in maternal pre-pregnancy weight, weight gain in pregnancy; and other characteristics may also have accounted for the observed increases in CD rates in Ghana. For example, the fear of childbirth, issues related to control and safety as well as risk associated with VD have been cited in previous studies as key reasons for CS [36]. However, the proportion is lower than what was found in Ghana by [14]. The possible reason for the inconsistency in study findings could be the study setting, the number of people that were used for the various studies and the years the studies were carried out as well as methodological designs. These findings imply that there should be encouragement of VD unless otherwise stated by a medical practitioner. Importantly, due to logistical constraints in most health facilities, especially in the rural areas of the country, it is equally prudent to raise clients’ and health professionals’ awareness about the adverse outcomes associated with CD and advantages of VD. Educating mothers about risks associated with CD and effective midwifery training could also help encourage mothers to deliver vaginally [1].

This study found a positive relationship between the odds of CD and age of mothers. Specifically, the odds of CD increased with their age. Those aged 45–49 had higher odds of CD compared to those aged 15–19. Findings of the current study, which involved data from a wider coverage of women across Ghana show evidence that advanced maternal age is a higher risk factor for CS and that the extent of the risk surges with advancement in maternal age, a finding that is corroborated in other studies [1, 11, 12, 28, 29, 37–39]. Berkowitz et al. [40] reiterated that advanced maternal age is generally believed to be associated with increased risk for adverse pregnancy outcome. Additionally, biological changes and complications (e.g., malposition, increased risk of hypertension, eclampsia, and diabetes) associated with aging pregnant women may heighten the risk of CD [41, 42]. Due to the increase in risk, some women voluntary opt for CD [43].

CD in this study was associated with the region of residence. Those in Upper East and Upper West regions had lower odds of CD compared to women in the Western Region. This finding is confirmed in other studies on the relationship between the geographical location and CD [1, 44]. Mothers staying in less developed regions are less likely to use CD services compared to those in regions that are more developed. The reason could be the easy access, availability and utilization of maternal healthcare facilities at private and public hospitals in the southern part of Ghana compared to the limited healthcare facilities in the Northern part of Ghana [14]. Additionally, there is better access to CD due to the high numbers of health facilities with the capacity to conduct CD in the souther part of Ghana [30].

There was an inverse relationship between parity and the odds of CD. Those with about 4 or more births had lower odds of delivering their babies through CS. This finding is consistent with previous research [13–15]. As explained by Manyeh et al. [14] on CD, women who might have undergone more than 1 CD do not get pregnant again to avoid further CD. Additionally, once the woman’s pelvis has been tested with a previous pregnancy and VD, subsequent deliveries vaginally tend to be less risky [15]. Hence, mothers who have had a lot of experiences with VD may be less likely to go in for CD.

Another significant finding in this current study was that mothers with female babies had lower odds of CD compared to those who had male children. From a socio-cultural perspective, the uncertainty attached to CD by most women of sub-Saharan descent due to its associated unpleasant experiences (e.g., physical, psychological and emotional pain/distress) suggest that the mothers with female babies may wish not to have CD because of a commonly held belief that such painful CD experiences might be transferred to their daughters later in life. Related to this households with a female as the head were more likely to go for CD compared to those headed by males, a finding similar to a Ghanaian community-based study [14]. What is unclear is whether this particular finding could be associated with the diverse sociocultural differences emanating from varied ethnic background of the women groups used for the study. It is therefore imperative for further studies to consider the association between the sex household head and CD to unearth the nuances.

### Strength and limitations of the study

By investigating caesarean outcomes of women from different cohort groups, this study realizes the arduous responsibility of discovering CD trends overtime among these diverse women groups. However, this study is not without limitations. First, the data did not capture any specific type of pregnancy complication that resulted in CD which could not help to ascertain whether performed CD was under medical indications (e.g., fetal mal-presentation) or based solely on maternal demand. Also, due to the cross-sectional nature of the study, causality could not be expressed between any of the independent variables and CD. Other limitations may include those commonly related to large database research such as alterations in the coding of procedures in charts, tables and other data abstraction errors. However, no considerable modifications were done during coding and extraction in the conduct of this study. Despite these limitations, this study provides evidence-based estimates on the prevalence of CD among women giving birth in health facilities in Ghana, as well as its associated factors.

## Conclusion and policy implications

In conclusion, women route of delivery is a potentially modifiable risk factor that are at two ends of the child delivery continuum (i.e., caesarean versus vaginal). The foregoing investigation of CD rates and associated factors as well as geographical differences would provide vital data for obstetric decision making on this medical intervention. The study findings show that the current CD in Ghana is approximately 18.5% which is above the WHO recommended proportion of 5–15%. The study also revealed strong associations between maternal age, region, parity, baby’s sex, and sex of household head and the probability of CD. Specifically, female babies are less likely to be delivered via CS whereas women with female househild head are more likely to deliver through CS. The high rates of CD noted in the current study do not essentially indicate good quality care or services. Health institutions with high CS rate should conduct comprehensive assessment of the associated factors toward obstetric care. Detailed medical justification for performing CS by doctors should also be provided to reduce the proportions of women opting for CD. Additionally, other quantitative and qualitative research ought to be conducted to better understand the socio-cultural beliefs, psychological factors and perceptions of Ghanaian women that may be contributing to the high uptake of CD in Ghana.

## Data Availability

Data is available on https://dhsprogram.com/what-we-do/survey/survey-display-437.cfm.

## Abbreviations

ANC: Antenatal Care
AOR: Adjusted Odds Ratio
CD: Caesarean Delivery
CI: Confidence Interval
CS: Caesarean Section
GDHS: Ghana Demographic and Health Surveys
MMR: Maternal Mortality Ratio
WHO: World Health Organization

## References

[1] Amjad A, Amjad U, Zakar R, Usman A, Zakar MZ, Fischer F (2018). Factors associated with caesarean deliveries among child-bearing women in Pakistan: secondary analysis of data from the demographic and health survey, 2012–13. BMC Pregnancy Childbirth.

[2] Asuquo EO, Orazulike NC, Onyekwere EC, Odjegba JN, Ojo AI, Ogbansiegbe JA (2016). Factors associated with preference for caesarean section among women in the ante-natal clinic of a tertiary hospital in the Niger Delta, Nigeria. A pilot study. J Adv Med Med Res.

[3] Betran AP, Ye J, Moller AB, Zhang J, Gülmezoglu AM, Torloni MR (2016). The increasing trend in caesarean section rates: global, regional and national estimates: 1990-2014. PLoS One.

[4] Harrison MS, Goldenberg RL (2016). Cesarean section in sub-Saharan Africa. Matern Health Neonatol Perinatol.

[5] Souza JP, Gülmezoglu A, Lumbiganon P, Laopaiboon M, Carroli G, Fawole B, Ruyan P, WHO Global Survey on Maternal and Perinatal Health Research Group (2010). Caesarean section without medical indications is associated with an increased risk of adverse short-term maternal outcomes: the 2004–2008 WHO Global Survey on Maternal and Perinatal Health. BMC Med.

[6] Babill S-P (2012). Lecture note “assisted delivery”.

[7] World Health Organization (2016). Provision of effective antenatal care: integrated management of pregnancy and childbirth.

[8] Ostovar R, Rashidian A, Pourreza A, Rashidi BH, Hantooshzadeh S, Ardebili HE, Mahmoudi M. Developing criteria for cesarean section using the RAND appropriateness method. BMC pregnancy and childbirth. 2010;10(1):1–8. 10.1186/1471-2393-10-52 PMC2949786 20840776

[9] Mekonen Y (2012). Maternal health in Ethiopia: challenges in achieving the MDG for maternal mortality: in-depth analysis of the EDHS 2000–2011.

[10] Kahsay S, Berhe G, Gebremari A (2015). Determinants of caesarean deliveries and its major indications in Adigrat hospital, Northern Ethiopia: a case-control study. Epidemiology.

[11] Al Rifai RH (2017). The trend of caesarean deliveries in Egypt and its associated factors: evidence from national surveys, 2005–2014. BMC Pregnancy Childbirth.

[12] Begum T, Rahman A, Nababan H, Hoque DM, Khan AF, Ali T, Anwar I (2017). Indications and determinants of caesarean section delivery: evidence from a population-based study in Matlab, Bangladesh. PloS One.

[13] Janoudi G, Kelly S, Yasseen A, Hamam H, Moretti F, Walker M (2015). Factors associated with increased rates of caesarean section in women of advanced maternal age. J Obstet Gynaecol Can.

[14] Manyeh AK, Amu A, Akpakli DE, Williams J, Gyapong M (2018). Socioeconomic and demographic factors associated with caesarean section delivery in Southern Ghana: evidence from INDEPTH network member site. BMC Pregnancy Childbirth.

[15] Mgaya AH, Massawe SN, Kidanto HL, Mgaya HN (2013). Grand multiparity: is it still a risk in pregnancy?. BMC Pregnancy Childbirth.

[16] Stanton CK, Holtz SA (2006). Levels and trends in cesarean birth in the developing world. Stud Fam Plan.

[17] Huang K, Tao F, Faragher B, Raven J, Tolhurst R, Tang S, Van Den Broek N (2013). A mixed-method study of factors associated with differences in caesarean section rates at community level: the case of rural China. Midwifery.

[18] Stanton C, Castro A, Adanu R, Heymann M, Adu-Bonsaffoh K, Lattof SR, Blanc A, Langer A (2013). Measuring coverage in MNCH: validating women’s self-report of emergency cesarean sections in Ghana and the Dominican Republic. PLoS One.

[19] World Health Organization (2010). World health statistics.

[20] Teguete I, Dolo A, Sissoko A, Thera A, Traore M, Djire MY, Mounkoro N, Dolo T, Traore Y. Determining factors of cesarean delivery trends in developing countries: lessons from point G National Hospital (Bamako-Mali). INTECH Open Access. 2012 May 23:161–202. Available from http://www.intechopen.com/books/cesarean-delivery/determining-factors-of-cesarean-deliverytrends-in-developing-countries-lessons-from-point-g-nat.

[21] Danso KA, Schwandt HM, Turpin CA, Seffah JD, Samba A, Hindin MJ (2009). Preference of Ghanaian women for vaginal or caesarean delivery postpartum. Ghana Med J.

[22] Seffah JD, Adu-Bonsaffoh K (2014). Vaginal birth after a previous caesarean section: current trends and outlook in Ghana. J West Afr Coll Surg.

[23] Prah J, Kudom A, Afrifa A, Abdulai M, Sirikyi I, Abu E (2017). Caesarean section in a primary health facility in Ghana: clinical indications and feto-maternal outcomes. J Public Health Afr.

[24] Ghana Statistical Service (GSS), Ghana Health Service (GHS), ICF International (2015). Ghana demographic and health survey 2014.

[25] Apanga PA, Awoonor-Williams JK (2018). Predictors of caesarean section in northern Ghana: a case-control study. Pan Afr Med J.

[26] Ghana Statistical Service (GSS) (2010). Ghana demographic and health survey, 2008.

[27] Bayrampour H, Heaman M (2010). Advanced maternal age and the risk of cesarean birth: a systematic review. Birth..

[28] Ghana Health Service (2011). Annual report.

[29] Rebelo F, Da Rocha CM, Cortes TR, Dutra CL, Kac G (2010). High cesarean prevalence in a national population-based study in Brazil: the role of private practice. Acta Obstet Gynecol Scand.

[30] Ghana Health Service (2016). Family health division annual report.

[31] Abebe FE, Gebeyehu AW, Kidane AN, Eyassu GA (2015). Factors leading to cesarean section delivery at Felegehiwot referral hospital, Northwest Ethiopia: a retrospective record review. Reprod Health.

[32] Singh P, Hashmi G, Swain PK (2018). High prevalence of cesarean section births in private sector health facilities-analysis of district level household survey-4 (DLHS-4) of India. BMC Public Health.

[33] Abu Anza SH, Abu Omar AA (2012). Frequency rate and indications of cesarean sections at Prince Zaid bin Al Hussein Hospital-Jordan. J R Med Serv.

[34] Dekker L, Houtzager T, Kilume O, Horogo J, van Roosmalen J, Nyamtema AS (2018). Caesarean section audit to improve quality of care in a rural referral hospital in Tanzania. BMC Pregnancy Childbirth.

[35] Menacker F, Declercq E, Macdorman MF (2006). Cesarean delivery: background, trends, and epidemiology. Semin Perinatol.

[36] Fenwick J, Staff L, Gamble J, Creedy DK, Bayes S (2010). Why do women request caesarean section in a normal, healthy first pregnancy?. Midwifery..

[37] Carolan M, Davey MA, Biro MA, Kealy M (2011). Older maternal age and intervention in labour: a population-based study comparing older and younger first-time mothers in Victoria, Australia. Birth.

[38] Hanif HM (2011). Association between maternal age and pregnancy outcome: implications for the Pakistani society. J Pak Med Assoc.

[39] Lin HC, Sheen TC, Tang CH, Kao S (2004). Association between maternal age and the likelihood of a cesarean section: a population-based multivariate logistic regression analysis. Acta Obstet Gynecol Scand.

[40] Berkowitz GS, Skovron ML, Lapinski RH, Berkowitz RL (1990). Delayed childbearing and the outcome of pregnancy. N Engl J Med.

[41] Luke B, Brown MB (2007). Elevated risks of pregnancy complications and adverse outcomes with increasing maternal age. Hum Reprod.

[42] Zgheib SM, Kacim M, Kostev K (2017). Prevalence of and risk factors associated with cesarean section in Lebanon—a retrospective study based on a sample of 29,270 women. Women Birth.

[43] Ecker JL, Chen KT, Cohen AP, Riley LE, Lieberman ES (2001). Increased risk of cesarean delivery with advancing maternal age: indications and associated factors in nulliparous women. Am J Obstet Gynecol.

[44] Nazir S (2015). Determinants of cesarean deliveries in Pakistan.

